# Editorial: Artificial intelligence for arrhythmia detection and prediction

**DOI:** 10.3389/fcvm.2025.1727758

**Published:** 2025-11-06

**Authors:** Panteleimon Pantelidis, Alexios Antonopoulos, Polydoros Kampaktsis, Evangelos Oikonomou

**Affiliations:** 1 3rd University Department of Cardiology, National and Kapodistrian University of Athens, Athens, Greece; 2 1st University Department of Cardiology, National and Kapodistrian University of Athens, Athens, Greece; 3 Division of Cardiology, Columbia University Irving Medical Center, New York, NY, United States

**Keywords:** arrhythmia, diagnostics, deep learning, artificial intelligence, risk prediction

The convergence of artificial intelligence (AI) and cardiovascular medicine has reached a critical juncture, offering unprecedented opportunities that can revolutionize arrhythmia care. This Research Topic presents ten pioneering articles that span from advanced detection algorithms to emerging prediction models, charting a clear trajectory toward truly predictive, personalized applications in cardiac arrhythmias.

## 1 Advanced detection technologies: achieving clinical excellence

He et al. introduce CardioAttentionNet (CANet), a portable deep learning (DL) model combining advanced architectures to classify five arrhythmia types with accuracies around: 97.37% ± 0.30% (normal), 98.09% ± 0.25% (supraventricular tachycardia), 92.92% ± 0.09% (ventricular tachycardia), 99.07% ± 0.13% (atrial fibrillation, AF), and 99.68% ± 0.06% (unclassified arrhythmias). AUCs exceed 99%, with 94.41% average accuracy on external data and 56.7 ms inference per electrocardiogram (ECG), demonstrating real-time feasibility. Complementing this, ECG-XPLAIM delivers a novel, explainable, inception-style model with multi-scale kernels (4, 20, 80 ms). Internal validation on MIMIC-IV and external on PTB-XL achieved AUCs of 0.88–0.99 and overall sensitivities/specificities >0.9. Grad-CAM visualizations offer insights into the model's predictions, bridging the gap between AI performance and clinical interpretability (Pantelidis et al.).

Summarizing relevant data, Yin et al.'s meta-analysis of heart rate variability (HRV)-based AF detection demonstrates a pooled sensitivity of 94% and specificity of 97%, with DL outperforming traditional machine learning, supporting AI readiness across diverse cohorts. Similarly, Papalamprakopoulou et al. review AI-enabled smartwatch AF detection, illustrating how continuous, noninvasive monitoring by wearable devices enhances preventive cardiology, while underscoring challenges in reducing false positives and integrating vast data streams into clinical workflows.

## 2 Emerging prediction capabilities: from risk assessment to personalized forecasting

Beyond accurate detection, contemporary AI is pivoting toward the more ambitious objective of forecasting arrhythmias and quantifying individualized risk. Zhang et al. propose a nomogram for sustained ventricular tachycardia in arrhythmogenic cardiomyopathy integrating age, sex, syncope, heart failure, echocardiographic parameters, and ECG findings. AUCs of 0.867 (training) and 0.815 (validation) mark significant strides in personalized risk stratification. Ingelaere et al. assess AI-enabled QRS fragmentation detection from 12-lead ECGs in 1,242 patients with implantable cardioverter-defibrillator (ICD) using support vector machines and features from phase-rectified signal averaging and variational mode decomposition. Despite a sensitivity of 0.76 and specificity of 0.92 for fragmentation, no significant link to ICD therapy or mortality was found, revealing limitations when translating traditional ECG markers into AI-based risk predictors. Liang et al.'s neural network for exercise stress ECG achieved AUC 0.83, sensitivity 0.89, and specificity 0.60 for significant coronary artery disease (CAD) detection, processing ECG segments, and clinical features. Results are delivered within one minute post-test, spotlighting the potential to streamline diagnostic workflows.

## 3 More clinical, molecular and multimodal applications

Antoun et al. provide a comprehensive scoping review of AI in AF across the full care continuum, emphasizing its value for identifying asymptomatic AF, tailoring therapy, and confronting key implementation barriers such as transparency, data integration, and regulatory alignment. Banerjee discusses mobile health AI technologies, detailing smartphone-based ECG and photoplethysmography (PPG) systems that democratize long-term rhythm monitoring, especially in underserved regions, and emphasizing accessibility alongside diagnostic accuracy. From a molecular viewpoint, Teng and Deng explore shared biology between AF and chronic kidney disease, identifying co-expressed genes (PPBP, CXCL1, RSAD2) and transcription factors (FOXC1, FOXL1, GATA2); this bioinformatics pipeline illustrates how AI can reveal mechanistic targets and candidate biomarkers, advancing precision therapeutics and risk stratification at the genotype-phenotype interface.

## 4 Current landscape and future directions

AI-driven detection systems for arrhythmias can now rival expert cardiologists. CANet and ECG-XPLAIM report sensitivities and specificities above 94%, and short inference times per ECG segment, enabling real-time deployment on portable and wearable devices. A meta-analysis of DL, HRV-based AF detection confirms the impressive performance across diverse cohorts. These advances support population-level screening via smart devices, lowering barriers to early AF identification (1).

By contrast, AI-based prediction models remain an emerging frontier. Current algorithms for ventricular tachycardia risk and post-exercise CAD detection achieve AUCs of 0.81–0.87. These models integrate clinical and lab test parameters, yet seem to lack robust external validation. Similarly, AI-detected QRS fragmentation shows reasonable detection accuracy, but no correlation with ICD therapy or mortality, highlighting the challenge of converting traditional ECG markers into prognostic tools. To achieve clinical maturity, prediction models could be enhanced by integrating multimodal data across large, diverse cohorts with longitudinal follow-up (2). Additionally, novel network architectures further drive advances in cardiac AI: Self-attention, applied to sequential ECG data, can capture long-range temporal dependencies and spotlight clinically salient signal segments. Even the more traditional, inception-style designs seem to work exceptionally well, enabling multiscale feature extraction and enhancing detection robustness. Depthwise separable convolutions and MobileNet-inspired modules ensure computational efficiency on constrained hardware (Pantelidis et al., Banerjee, Teng and Deng, 3).

However, critical hurdles remain. Explainability is essential for clinician trust and regulatory approval; Grad-CAM visualizations are increasingly integrated to reveal model decision pathways. Data heterogeneity, concerning varying ECG hardware, sampling rates, and annotation standards, limits generalizability and demands rigorous standardization. Interoperability across electronic health records and wearable platforms requires harmonized data formats. Moreover, evolving regulatory frameworks for AI as a medical device necessitate transparent validation, real-world performance monitoring, and post-market surveillance to detect algorithmic drift and bias (3–5).

The next frontier lies in multimodal integration: combining continuous ECG, imaging, genomic, and environmental data for comprehensive risk modeling. Agentic AI could autonomously adjust monitoring and therapeutic recommendations in real time, optimizing care pathways. Transformer-based architectures tailored to multidimensional cardiac data may predict imminent arrhythmic events by detecting subtle premonitory signal shifts. Digital twin frameworks, enabling virtual patient models continuously updated with live data, could simulate arrhythmic risk under different interventions, enhancing personalized preventive strategies (6). Realizing predictive, personalized arrhythmia care will require sustained multidisciplinary collaboration among cardiologists, data scientists, bioengineers, and ethicists. By addressing explainability, data integration, and equitable access, AI-enabled prediction can transition from research prototypes to life-saving clinical tools, heralding a new era of proactive cardiovascular medicine.
